# A novel chronic hepatitis B mouse model with immune activation and liver fibrosis

**DOI:** 10.1128/spectrum.02513-24

**Published:** 2025-07-24

**Authors:** Di Wu, Zihan Liu, Zhe Song, Leiyu Yu, Zhenzhen Guan, Huanfei Liu, Jin Zhao, Zhongzhen Jin, Xugang He, Yaqing Zhang, Lanxiao Cao, Guifu Dai, Jun Huang, Qiaozhen Kang

**Affiliations:** 1 Department of Biotechnology, School of Life Sciences, Zhengzhou University 12636, Zhengzhou, China; 2 Zhengzhou Key Laboratory of Development and Application of Biological Resources and New Drug Creation, Zhengzhou University 12636, Zhengzhou, China; University of Manitoba, Winnipeg, Canada

**Keywords:** mouse model, liver fibrosis, chronic hepatitis B, porcine serum, pAAV-HBV1.2 plasmid, viral persistence

## Abstract

As one of the key features of the immune active (IA) phase (HBeAg-positive) in chronic hepatitis B (CHB), liver fibrosis (LF) is a critical treatable stage to avoid progressing to hepatocellular carcinoma (HCC). There is an urgent need to establish mouse models that mimic CHB during this phase to clarify the mechanisms underlying hepatitis B virus (HBV) clearance and liver injury, as well as to facilitate therapy development. In the present work, we introduce a novel HBV mouse model via injections of porcine serum (PS, an immunological liver injury inducer), in combination with hydrodynamic injection of pAAV-HBV1.2 plasmid. The (pAAV-HBV1.2+PS BALB/c) mouse model we developed exhibits the typical features of the IA phase in CHB, including high/intermediate serum HBsAg levels, positive HBeAg and HBV DNA, intermittent elevation of serum alanine aminotransferase, and significant LF. Notably, despite dramatic increases in both duration and intensity of HBV viremia, PS induced definitive LF within 12 weeks only in BALB/c, not in C57BL/6J mice. The heightened functional adaptive immunity impairment, characterized by increased PD-1/PD-L1 signaling, may contribute to the improvement in HBV persistence, whereas intermittent HBV activation, followed by liver injury induced by PS injection, primarily drives LF progression. Furthermore, we validate the characteristics of this model by treating the model with entecavir (an HBV replication blocker). Consequently, we have established a novel CHB mouse model in the IA phase, which undoubtedly holds significant value for deepening our understanding of CHB progression mechanisms and evaluating corresponding therapies.

**IMPORTANCE:**

Here, we constructed a novel mouse model which shows the typical features of IA phase chronic hepatitis B, including positive serum HBV-indicators (hepatitis B surface antigen, HBsAg; hepatitis B e antigen, HBeAg and HBV DNA), intermittent serum alanine aminotransferase elevation, and significant LF. This model could be a valuable platform for understanding the mechanisms associated with virus clearance, liver damage, immune tolerance, and immune activation, and for the development of therapies. The PS injections can remarkably attenuate the clearance of HBV in pAAV-HBV1.2 mice (BALB/c and C57BL/6J), but only in HBV carrier BALB/c mice induce moderate/severe LF. Impairment of adaptive immunity by PS injections accounts for the model’s HBV-persistence improvement, whereas the intermittent HBV activation and immunological liver injury by each PS injection in pAAV-HBV1.2 mice are responsible for LF. Blockage of HBV replication can diminish LF progression. These findings provide new insights into the study of interactions between immune responses and the virus.

## INTRODUCTION

Although the availability of efficient vaccines, hepatitis B virus (HBV) infection-related diseases remain a significant global healthcare issue (1, 2). Current therapeutic options for chronic hepatitis B (CHB) can effectively reduce viral load but not achieve a functional cure (3, 4). CHB patients still have a high risk of progression to liver fibrosis (LF), cirrhosis, and hepatocellular carcinoma (HCC) (5). HBV exhibits minimal cytotoxicity, and the adaptive immunity that changes from immune tolerance to progressive immune activation, reactivation, and exhaustion is crucial for the progress of CHB (6).

Four phases of CHB have been defined on levels of viral replication and dynamics in liver disease progression: immune tolerant (IT), immune active (IA), inactive carrier (IC), and hepatitis B e antigen (HBeAg)-negative hepatitis (ENEG). Studies of these clinically relevant phases help us gain a better understanding of the global picture of CHB. Earlier research on CHB in different phases mainly focused on the immune and virological measurements in the blood, due to the fact that liver biopsy samples from patients are difficult to acquire. However, it is important to study the hepatic viral and immune characteristics in CHB patients at different periods, since immune dynamics differ in the blood and liver. Although recent studies have comprehensively illustrated the cellular and immune characteristics of the liver during various stages of CHB, the precise underlying mechanisms remain elusive (7, 8). The IA phase is characterized by hepatitis, LF, and fluctuating serum alanine aminotransferase (ALT) and viremia (9, 10), renamed HBeAg-positive CHB, which is considered to be a more accurate description of the phase’s characteristics, by the European Association for the Study of the Liver (EASL) (11, 12). IA is an important phase in the development of CHB and a major contributor to related liver damage. Nevertheless, the characteristics and specific mechanisms for the formation of this phase in CHB are still not fully understood, due to the lack of animal models that can accurately represent it.

Previously, several highly valuable HBV carrier mouse models have been established (13–16). Transgenic mice, for example, have been widely used to understand virus replication and virus-host interactions (15). However, research on HBV rebound and the related hepatic injury in these mice is limited as a result of their inherent immune tolerance (17). Although HBV mouse models involving LF can be constructed by introducing a hepato-toxin into HBV carrier mice, the descriptions of these composite models regarding HBV indicators, immunological characteristics, the causes and the degree of LF, as well as the transcriptomic features are somewhat lacking in comprehensiveness in specifying which period of these models in the natural history of chronic HBV infection (13, 18, 19). Moreover, the contribution of the virus and the immune system to these HBV-related fibrosis models remains largely unresolved. These issues are the possible reasons why such models have not been widely applied since they were first introduced. The dually humanized mice with liver and hematopoietic components have been developed (20), with immunopathological characteristics of CHB (inflammation, liver fibrosis, and cirrhosis). However, due to the mismatches in the human leukocyte antigen (HLA) system between the xenograft and the host, caused by engraftment of xenogeneic liver and hematopoietic system, there are still concerns over whether the nature of immune recognition and reactions against the virus in such a system is in line with that of natural infection (21). In addition, the experimental complexity, limited human cell accessibility, and ethical issues are unavoidable at present.

Currently, various immunocompetent mouse models by injecting vectors containing 1.2- or 1.3-fold over-length HBV genomes via hydrodynamic injection (HI) are widely used as they can induce HBV-specific immunoreactions (22, 23). However, it is difficult to cause a sufficient immunoreaction and sufficient pathological processes, though several mouse models exhibit longer HBV persistence than the classical C57BL/6J mice and/or slight LF development (15). Porcine serum (PS) is widely used to trigger recurrent immune reaction, thereby inducing immunological LF in rats. This has been proposed as a substitute for HBV-associated LF to be used for developing drugs for chronic hepatitis B-related liver fibrosis (CHB-LF) (24, 25). Therefore, we hypothesize that it is possible to create a mouse model with features of the IA phase CHB via PS and 1.2-fold over-length HBV genome injections into an immunocompetent mouse. It remains unclear whether injections of PS can sustain HBV persistence and induce considerable immune injury in mice. To ascertain this, in the present study, the effects of PS injections on LF progress and HBV persistence in C57BL/6J (HBV-persistence strain) and BALB/c (HBV-clearance strain) mice pre-received pAAV-HBV1.2 plasmids were investigated (22). The characteristics and the underlying mechanisms of our model were addressed. The similarity of our mouse model to human CHB-LF was further proved via blockage experiments of HBV replication. Together, this novel mouse model is suitable to study HBV immunopathology, especially the host-HBV interactions during LF progression, and to assess the corresponding therapies.

## RESULTS

### Improvement of HBV persistence in both strains of HBV + PS mice

**Fig 1:**
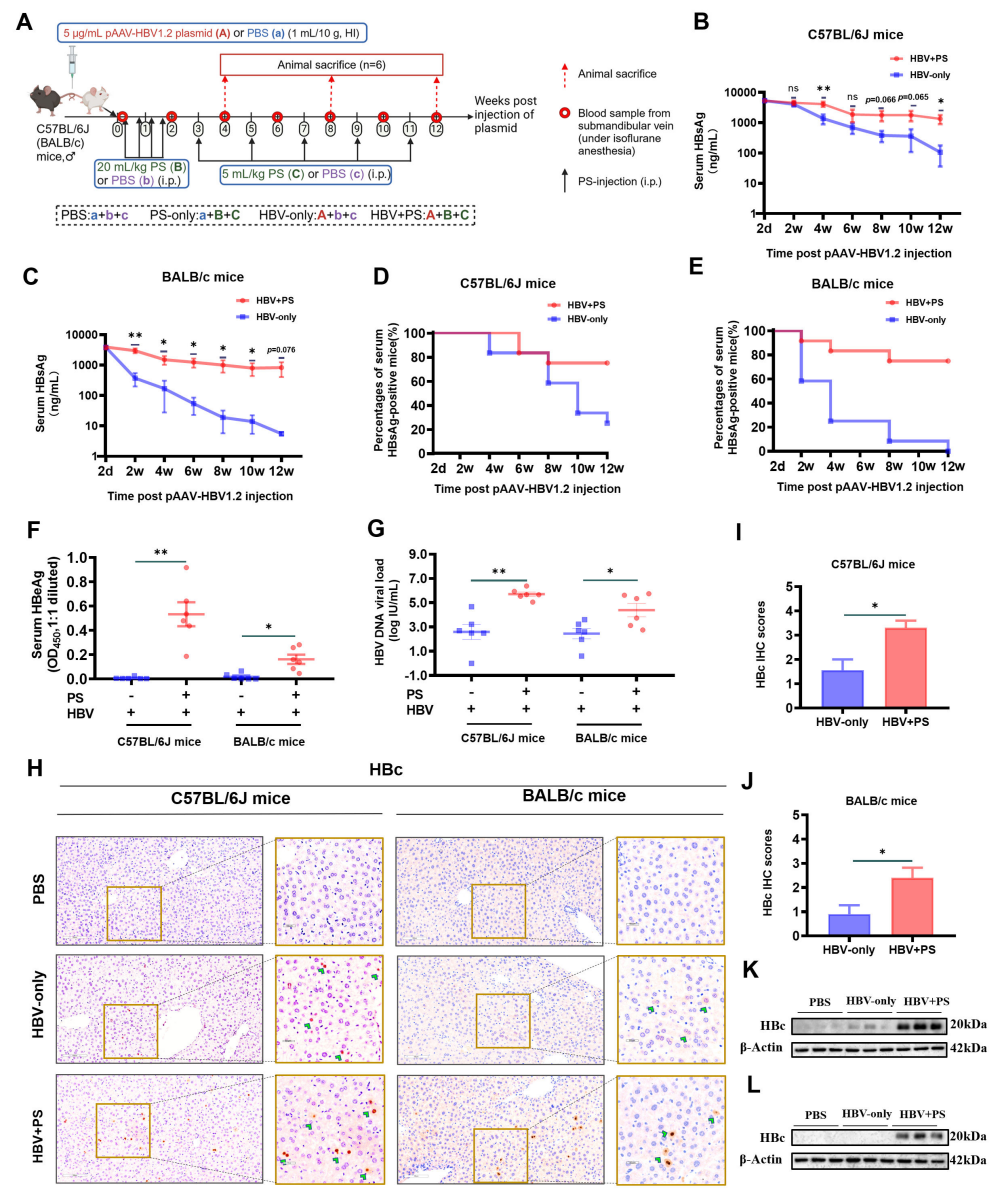
HBV persistence improvement by repeated injections of PS in both strains of mice. (**A**) Schematic diagram of the animal experiments. (**B, C**) Serum HBsAg in mice was assayed by the ELISA method, and (**D, E**) the percentages of serum HBsAg positive (≥10 ng/mL) were compiled statistics (*n* = 12). (**F**) Serum HBeAg and (**G**) HBV DNA were assayed by ELISA and quantitative PCR (qPCR), respectively (12 wpi, *n* = 6). Hepatic HBc proteins were assayed by (**H–J**) immunohistochemistry (IHC) staining (*n* = 4) and by (**K-L**) western blotting (WB) (*n* = 3), respectively; for the representative images of IHC (**H**), the left panels, 200×; scale bar = 100 µm; the right panels, 400×; scale bar = 50 µm. All quantitative results are presented as mean ± SEM. *P < 0.05, **P < 0.01.

As after receiving pAAV-HBV1.2 plasmid, C57BL/6J mice exhibit an HBV-persistence phenotype, while BALB/c mice exhibit an HBV-clearance phenotype (22), we selected these two mouse strains to explore the effects of intraperitoneal PS injections on HBV persistence and liver injury in mice pre-received pAAV-HBV1.2 plasmid (Fig. 1A). In accordance with the previous research (22), after hydrodynamic injection (HI) of pAAV-HBV1.2 plasmid in BALB/c mice, serum hepatitis B surface antigen (HBsAg) rapidly decreased to a very low level within 4 wpi, whereas it in C57BL/6J mice gradually declined (Fig. 1B and C). However, PS injections increased HBV persistence in both mouse strains. The serum HBsAg in HBV+ PS mice was sustained (4–12 wpi), and it was higher in HBV + PS mice than in the HBV-only mice.

In HBV + PS mice (12 wpi), 75% (9/12 mice) of the mice remained HBsAg positive, while in HBV-only mice, the time for HBsAg levels to decrease by half was, respectively, about 4 weeks (BALB/c) and 8 weeks (C57BL/6J) (Fig. 1D and E). Correspondingly, in both mouse strains, serum HBeAg (Fig. 1F), HBV DNA (Fig. 1G), and hepatic HBc (Fig. 1H through L) were each higher in the HBV + PS mice than in the HBV-only (*P* < 0.05). This shows HBV persistence in both mouse strains was strongly enhanced by PS injections, and HBV + PS BALB/c mice were converted to an HBV persistence phenotype. Moreover, the levels of serum HBV markers of the mice (HBV + PS) align with the definition of IA phase CHB in clinical practice guidelines (11, 12).

### Remarkable liver inflammation, injury, and fibrosis progress caused by injections of PS in pAAV-HBV1.2 BALB/c mice

**Fig 2:**
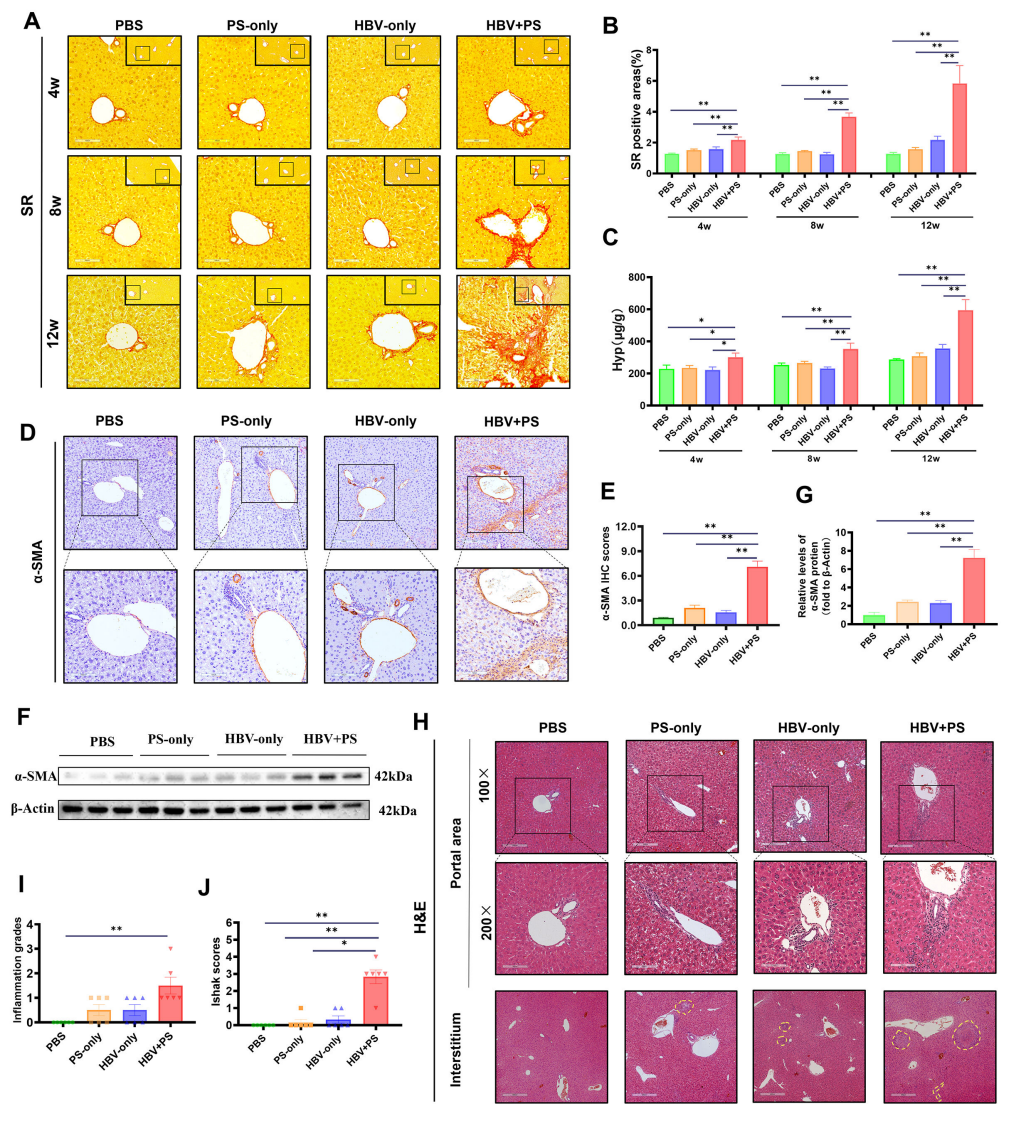
Progressed liver inflammation, injury, and fibrosis caused by injections of PS in pAAV-HBV 1.2 BALB/c mice. Levels of collagen deposition were appraised by (**A, B**) Sirius red (SR) staining for liver sections, and by (**C**) the quantification of hepatic hydroxyproline (Hyp, *n* = 6); for the representative images of SR staining, 200×, scale bar = 100 µm; the top-right corner, 100×; scale bar = 200 µm. Hepatic α-SMA protein levels were analyzed by (**D, E**) IHC (*n* = 4) and by (**F, G**) WB (*n* = 3); for the representative IHC micrographs, the upper panels, 100×; scale bar = 200 µm; the lower panels, 200×; scale bar = 100 µm. The pathological changes in the liver were observed through (**H**) H&E staining (12 wpi), and (**I**) the liver inflammation grades and (**J**) the fibrosis stages were, respectively, assessed by the Scheuer system and Ishak scoring system, respectively. For the representative images of H&E staining, portal area: the upper panels, 100×; scale bar = 200 µm; the lower panels, 200×; scale bar = 100 µm; interstitium: 50×; scale bar = 400 µm. All data are presented as mean ± SEM. *P < 0.05, **P < 0.01.

Evident LF is also one of the key pathological characteristics of the IA phase in CHB (11, 12). Therefore, the LF severity in various groups of the two strains of mice was subsequently assessed (Fig. 1A and 2; Fig. S1). Clearly, moderate/severe LF progression only appeared in HBV + PS BALB/c mice, not in other BALB/c or C57BL/6J mice (Fig. 2A through C; Fig. S1A through C). In HBV + PS BALB/c mice, the mean percentages of Sirius red-stained areas increased over time to 2.18% (4 wpi), 3.68% (8 wpi), and 5.84% (12 wpi), which were significantly higher than in the other BALB/c mice at corresponding time points (Fig. 2A and B, *P* < 0.01). A similar conclusion can be obtained by the hydroxyproline levels (Fig. 2C, *P* < 0.05).

As activated hepatic stellate cells (aHSCs) play crucial roles in LF development, the hepatic protein expressions of alpha-smooth muscle actin (α-SMA, a key marker of aHSCs) were appraised by immunohistochemistry (IHC) staining and western blotting (12 wpi). Alpha-SMA expression was much stronger than the other three groups observed in the HBV + PS BALB/c mice (*P* < 0.01) (Fig. 2D through G). Correspondingly, hepatic histopathological changes (hematoxylin and eosin [H&E] staining) showed there was remarkably severe inflammatory cell infiltration and necrotic foci in the HBV + PS BALB/c mice, with a higher inflammation score than in the PBS mice (*P* < 0.01, Fig. 2H and I) and a higher Ishak fibrosis score than the other three groups (*P* < 0.05) (Fig. 2J). This indicates that BALB/c mice are more prone to immunological liver injury than C57BL/6J mice, though the latter exhibit stronger HBV persistence, and PS injections only in BALB/c mice pretreated with pAAV-HBV1.2 plasmid successfully induce LF.

### Intermittent elevation of serum HBsAg, cytokines, and ALT in the HBV + PS BALB/c mice accompanied by the PS injection

**Fig 3:**
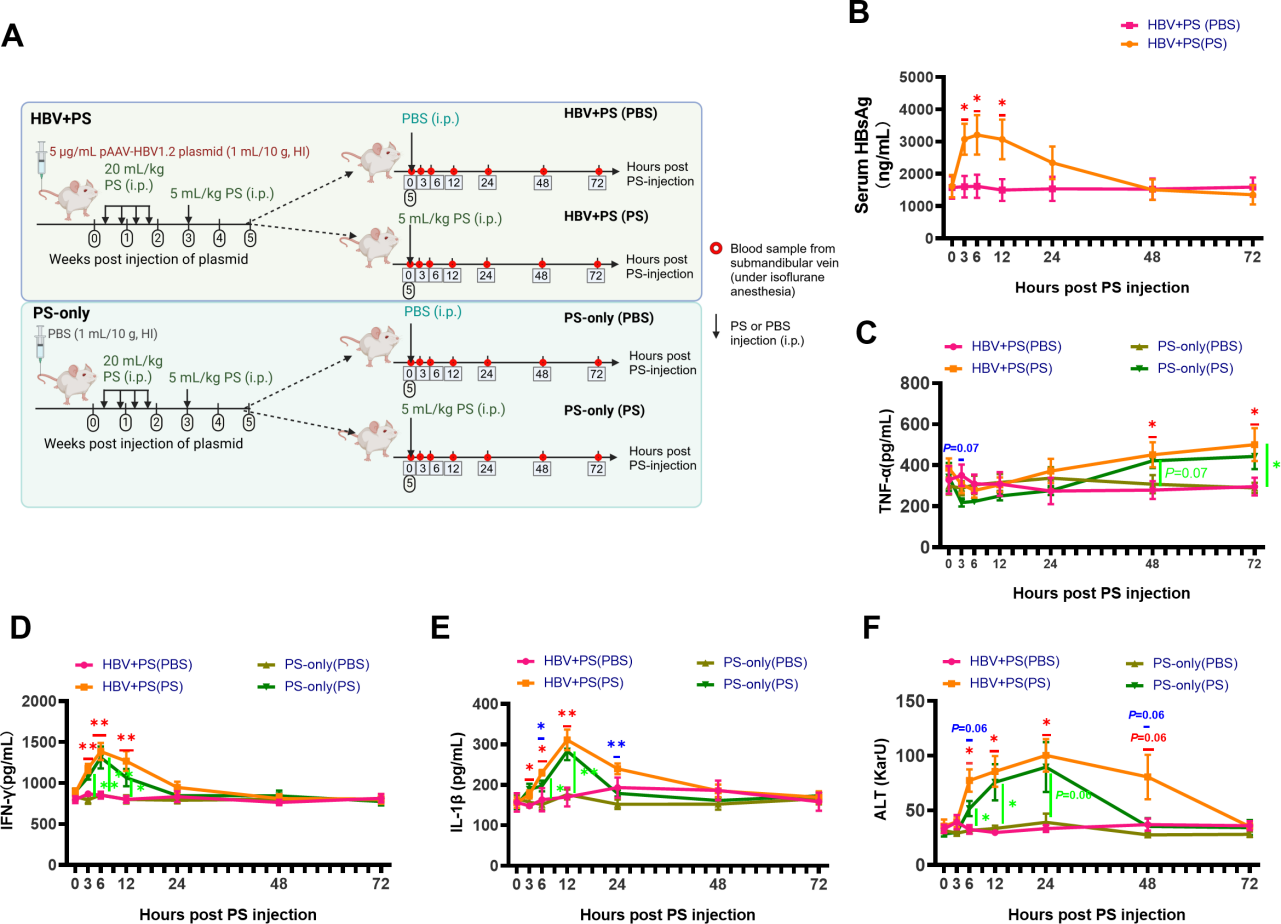
Immune activation and liver injury in the model mice by PS injection. (**A**) Schematic diagram of immune activation and liver injury observation. (**B**) Serum HBsAg, (**C**) TNF-α, (**D**) IFN-γ, (**E**) IL-1β, and (**F**) ALT were quantified from 0 to 72 h after PS injection (5 wpi). All data are presented as mean ± SEM. *P < 0.05 and **P < 0.01 (in red) for HBV + PS (PS) vs. HBV + PS (PBS); *P < 0.05 and **P < 0.01 (in green) for PS-only (PS) vs. PS-only (PBS); *P < 0.05 and **P < 0.01 (in blue) for HBV + PS (PS) vs. PS-only (PS).

Changes in serum HBsAg, cytokines including tumor necrosis factor-alpha (TNF-α), interferon gamma (IFN-γ), interleukin-1 beta (IL-1β), and ALT accompanied by PS injection were detected in real time (0–72 h) (Fig. 3A). In the HBV + PS mice while receiving PS, HBsAg sharply increased as early as 3 h post-injection of PS (hpi) (vs. PBS injection, *P* < 0.05), peaking at 6 hpi and then decreasing from 12 hpi (vs. PBS injection, *P* < 0.05; Fig. 3B), whereas TNF-α gradually decreased after PS injection (3–12 hpi), and then slowly returned to near baseline at 24 hpi, followed by gradually increasing from 48 hpi (vs. PBS injection, *P* < 0.05; Fig. 3C).

Levels of IFN-γ (Fig. 3D) and IL-1β (Fig. 3E) showed similar trends to HBsAg, increasing from 3 hpi (vs. PBS injection, *P* < 0.05), but peaking at 6 and 12 hpi (vs. PBS injection, *P* < 0.05), respectively.

ALT, another key marker of IA phase in CHB, increased from 6 hpi, peaking at 24 hpi (vs. PBS injection; 6–24 hpi, *P* < 0.05), and returned to baseline (0 hpi) within 72 hpi (Fig. 3F). Notably, after PS injection in the PS-only mice, serum ALT and cytokines of IFN-γ and IL-1β exhibited similar trends to the HBV + PS mice, but with smaller amplitudes of elevation and shorter durations (Fig. 3D, E and F).

### Adaptive immune exhaustion in HBV + PS BALB/c mice

**Fig 4:**
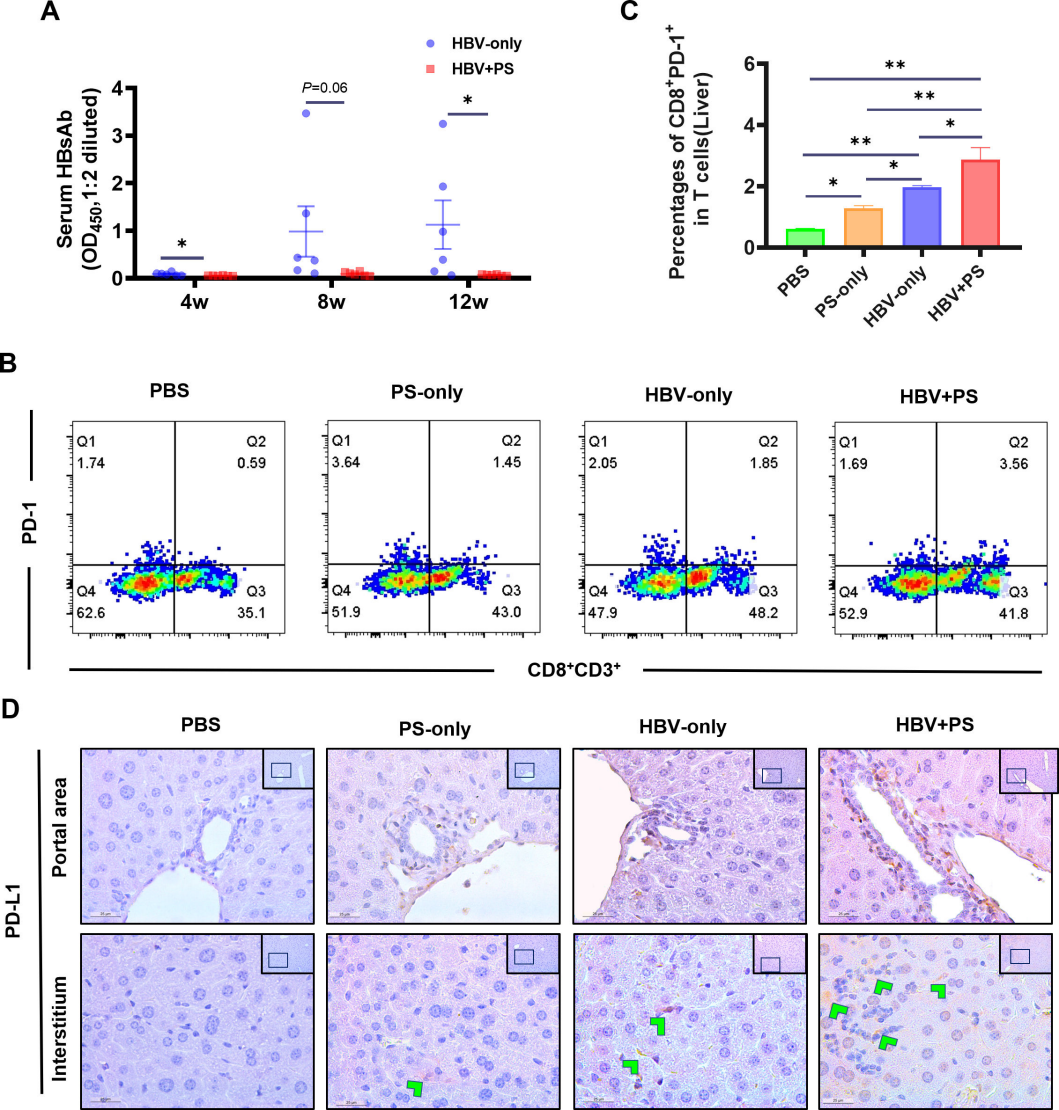
Functional impairment of adaptive immunity in HBV + PS BALB/c (the model) mice. (**A**) Serum HBsAb was assayed by the ELISA method (*n* = 6). (**B**) Representative flow cytometry plots and gating strategy showing relative frequencies and (**C**) the statistics of the percentages of CD8^+^PD-1^+^ T cells (12 wpi, *n* = 3). (**D**) Representative micrographs (IHC) showing hepatic PD-L1 proteins (400×, scale bar = 25 µm; the top-right corner, 200×; scale bar = 100 µm). All data are presented as mean ± SEM. *P < 0.05, **P < 0.01.

As the chronicity of HBV infection is thought to be the result of impaired HBV-specific immune responses that cannot efficiently eliminate or cure the infected hepatocytes (26), serum hepatitis B virus surface antibody (HBsAb) in BALB/c mice was detected (Fig. 4A). In HBV-only mice, it sharply increased from 4 to 8 wpi, and then remained at the highest level until 12 wpi. However, it stayed very low from 4 to 12 wpi in the model mice (HBV + PS mice) (vs. HBV-only, *P* < 0.05). Considering that the programmed cell death protein 1 (PD-1)/programmed cell death ligand 1 (PD-L1) signaling is thought to be crucial in inhibiting HBV-specific CD8^+^ T cells during chronic HBV infection (27), negatively regulating T-cell activation and anti-HBV cytokine production (28, 29), we explored the state of PD-1/PD-L1 signaling (Fig. 4B through D). The proportion of CD8^+^PD-1^+^ T cells among hepatic mononuclear cells was significantly higher in model mice than in mice in other groups (*P* < 0.05, Fig. 4B and C). Similar results were observed in hepatic cells with PD-L1^+^ (Fig. 4D). These indicate that PS injection in HBV carrier mice can effectively promote functional impairment of adaptive immunity and thereby attenuate HBV clearance.

### Attenuation of LF progression in model mice by blocking HBV replication

**Fig 5:**
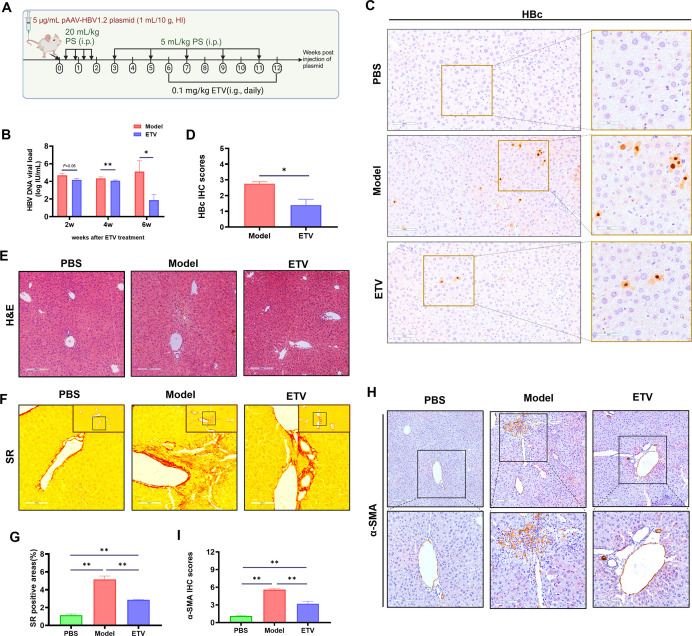
Attenuation of liver fibrotic formation in model mice via HBV replication inhibition by entecavir. (**A**) Experiment scheme showing ETV treatment for 6 weeks from 6 wpi. (**B**) HBV DNA titers based on qPCR after ETV treatment for 2, 4, and 6 weeks, *n* = 6. (**C**) Representative micrographs (IHC) showing the HBc proteins and (**D**) the statistical analysis. (**E**) Representative images of H&E-stained liver sections showing pathological changes after ETV treatment for 6 weeks (100×, scale bar = 200 µm). (**F**) Representative images of SR-stained liver sections (200×, scale bar = 100 µm; the top-right corner, 100×; scale bar = 200 µm) and (**G**) the statistical analysis (*n* = 6). (**H**) Representative micrographs (IHC) showing α-SMA proteins and (**I**) the statistical analysis (*n* = 4). All data are presented as mean ± SEM. *P < 0.05, **P < 0.01.

To explore whether HBV replication is essential for the CHB-LF features exhibited in the model mice, we treated the model mice with entecavir (ETV, an HBV inhibitor) (Fig. 5A). Serum HBV DNA and hepatic HBc protein were greatly decreased after treating the model mice with ETV for 6 weeks, compared to that in the un-treatment (*P* < 0.05, Fig. 5B through D); while the inflammatory cell infiltration was lightened (Fig. 5E), the collagen deposition and α-SMA expression were attenuated (*P* < 0.01, Fig. 5F through I). These indicate that HBV replication itself is crucial for LF progress in the model mice.

## DISCUSSION

HBV persistence in the hydrodynamic injection (HI) mouse model is affected by various factors such as mouse strain, age, sex, plasmid skeleton, and plasmid dose (14, 15). Viremia in C57BL/6 mice receiving the pAAV-HBV1.2 plasmid could be sustained for 24–36 weeks; whereas in BALB/c mice, it is usually cleared within 3–5 weeks. Lack of adequate immune response (C57BL/6) or acute immune clearance of the virus (BALB/c) makes it difficult for such models to achieve satisfactory pathological progression to LF (22).

**Fig 6:**
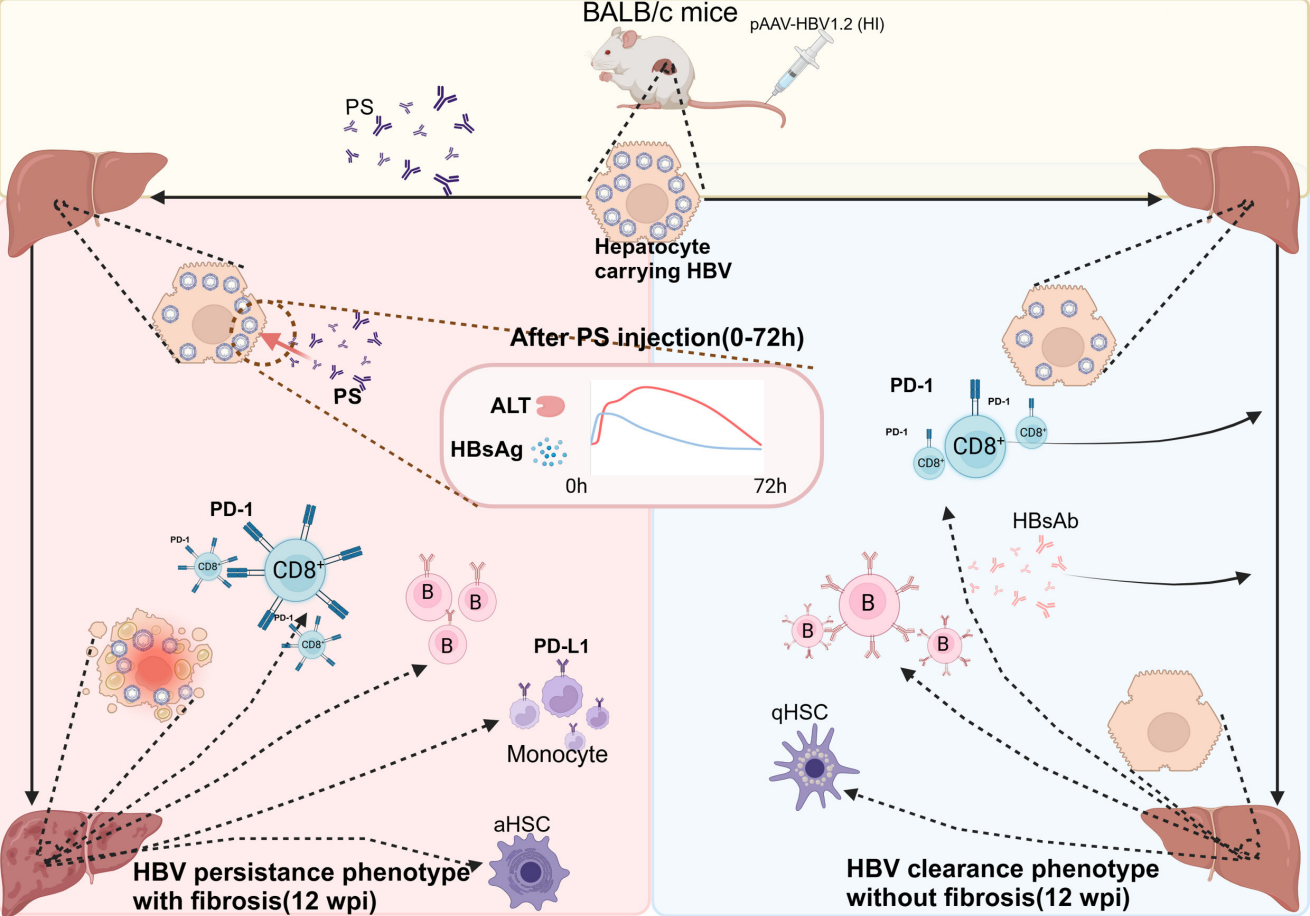
Graphic summary of the model characteristics.

In the present study, we established a mouse model with features of IA phase CHB (HBeAg-positive CHB), exhibiting persistent HBV replication and progressive immune histopathological changes, through repeated PS injections in the pAAV-HBV1.2-pretreated (HI) BALB/c mice (Fig. 1 to 3). This model exhibits the following remarkable characteristics (Fig. 6): (i) 75% (9/12 mice) of the model mice can maintain HBV persistence at high levels (HBeAg-positive, HBV DNA-positive, and HBsAg-positive) for longer than 12 weeks, and 5/6 of the mice can progress to Ishak stage ≥3 (LF) within 12 wpi. (ii) Long-term persistent HBV replication and intermittent HBV activation caused by PS injection are essential for CHB-LF progression; pAAV-HBV1.2 and PS injections establish and sustain a microenvironment in the liver by driving functional exhaustion of CD8^+^ T cells and reducing anti-viral antibody production. (iii) LF progression in model mice was mainly triggered by liver injury induced after each PS injection, with HBV itself being a necessary factor in this progression. This process was characterized by intermittent HBV activation and followed by serum ALT elevation, severe hepatic inflammation, and liver injury, thereby accelerating HSC activation and collagen deposition.

In pAAV-HBV1.2 (HI) BALB/c mice, HBV was more prone to be cleared than in C57BL/6J mice (Fig. 1), which is in line with the previous report (22). However, importantly, PS injections make both strains of mice sustain HBV at higher levels, with the model mice even exhibiting longer HBV persistence when compared to those of C57BL/6J mice (HBV-only). Impairment of the adaptive immune response is the key factor that prevents chronic HBV infection from being functionally cured (26). Consistently, our results reveal that PS injections in the HBV carrier mice greatly enhance serum HBsAg (Fig. 1A through C) and decrease serum HBsAb (Fig. 3A). In chronic HBV infection patients and HBV carrier mice and macaques, the interaction between the inhibitory receptor PD-1 on lymphocytes and its ligand (PD-L1) plays a critical role in T-cell exhaustion by inducing T-cell inactivation (26–29). Anti-PD-1 therapy may decrease or entirely clear HBsAg in CHB patients (27, 28). The proportion of CD8^+^PD-1^+^ T cells in hepatic mononuclear cells and the number of PD-L1^+^ cells in liver sections were increased (Fig. 4B through D), indicating that exhaustion of adaptive immunity, at least partly, underlies the increase in HBV persistence; meanwhile, the production of HBsAb is also damaged, suggesting that adaptive immunity is impaired in this model.

Although cellular and humoral immune responses are unable to eradicate the HBV infection in CHB patients, they are sufficient to sustain low-grade liver inflammation (7). Mounting evidence shows that severe hepatitis can occur due to HBV reactivation (30–32), such as in chronic HBV carriers treated for hepatobiliary malignancies with radiotherapy, in individuals with HBV and HCV co-infection treated for HCV infection with direct-acting antivirals, and in chronic HBV carriers co-infected with HIV who have accelerated LF progression compared to individuals with HBV mono-infection (33). These findings indicate that disturbing the immune balance of chronic HBV carriers can cause HBV (re)activation. Interestingly, recent research on acute-on-chronic liver failure (ACLF) induced by combined PS with D-galactosamine and lipopolysaccharide showed prominent immune dysregulation with significant elevation of monocytes and macrophages, whereas adaptive immune-related cells were reduced (34).

Repeated PS injections to rats rather than to mice can induce immuno-hepatic fibrosis and even cirrhosis (35–37). The PS-induced LF rat model has been widely used for a long time to assess the effectiveness of drugs for chronic hepatitis (37). Our results showed that in PS-only and HBV-only BALB/c mice, there was only slight extracellular matrix deposition (Fig. 2), though ALT in PS-only mice was transiently increased after PS injection from 6 to 12 h (Fig. 3F). Moreover, the duration of elevated ALT levels in the mice receiving PS injections alone was shorter than in the model mice. These results suggest that PS is a potent accelerator of HBV-associated LF. Importantly, the fibrotic pathologic changes in model mice could be reversed by 6 weeks of ETV treatment (Fig. 5). Thus, in our mouse model, HBV itself is also a necessary condition. Furthermore, the significant therapeutic effect of ETV treatment in the model mice precisely reflects the necessity of long-term administering first-line nucleotide analog (NA) in patients in the IA phase, which aligns with clinical practice (12).

Taken together, we established a novel mouse model with features of the IA phase of chronic HBV infection. In the modeling, the persistence of HBV replication is essential, and PS injection can effectively accelerate LF progression. LF induced by PS injections probably accrued due to adaptive immune exhaustion, thereby causing an HBV viremia surge and creating a suitable immune and inflammatory microenvironment for LF development. This mouse model may provide new insights into the mechanisms underlying LF during chronic HBV infection and a platform for investigating the immune-related mechanisms underlying HBV clearance, persistence, and activation. Furthermore, the model offers a reliable and simple platform to assess anti-HBV drugs and drugs for treating CHB patients in the IA phase, which will facilitate the development of new therapeutics. Notably, we mainly focused on establishing a CHB mouse model and assessing its similarities with the IA phase of chronic HBV infection. However, the detailed immunopathological mechanisms in the model that are responsible for balancing HBV persistence and LF progression still need to be studied. In addition, in the modeling, the frequency and dose of PS injections are the key factors for realizing HBV persistence and LF progression, and for preventing mice from death due to hyper-immunity.

## MATERIALS AND METHODS

### Plasmid and drugs

The pAAV-HBV1.2 plasmid was kindly provided by Prof. Pei-Jer Chen (National Taiwan University, Taipei, China). Porcine serum (PS, 26250084) was provided by Gibco BRL-Life Science Technologies, Inc. (Grand Island, NY, USA). Carboxymethylcellulose sodium (CMC-Na) was obtained from Anhui Sunhere Pharmaceutical Excipients Co., Ltd. (Anhui, China). Entecavir (ETV) was supplied by Chia Tai Tianqing Pharmaceutical Group Co., Ltd. (Nanjing, China).

### Mice

Male mice (C57BL/6J and BALB/c) aged 6–8 weeks were obtained from Hunan SilaikeJingda Experimental Animal Co., Ltd. All animals were maintained under a specific pathogen-free (SPF) condition in a 12 h light/dark cycle with free access to food and water. The animal experiments were performed following “Regulations for the Administration of Affairs Concerning Experimental Animals,” which are the national guidelines for the care and use of animals in scientific research.

### Animal grouping and modeling

For studies of HBV persistence and LF progression, mice were divided into four groups (PBS, PS-only, HBV-only, and HBV + PS). The mice were treated via hydrodynamic injection (HI) with 5 µg/mL of pAAV-HBV1.2 plasmid (1 mL/10 g; HBV-only and HBV + PS) or the same volume of phosphate-buffered saline (PBS) (1 mL/10 g; PBS and PS-only), and then were intraperitoneally injected (i.p.) with PS (PS-only, HBV + PS) or PBS (PBS, HBV-only) under the protocol shown in Fig. 1A.

For studies of HBV activation and immunological liver injury induced by PS, BALB/c mice were divided into four groups, including HBV + PS (PBS), HBV + PS (PS), PS-only (PBS), and PS-only (PS). Similar to the modeling procedure shown in Fig. 1A, mice pre-received injection of pAAV-HBV1.2 plasmid (the two HBV + PS groups) or injection of the same volume of PBS (the two PS-only groups) were all treated with PS (i. p.) till 4 weeks after the injection of plasmid (4 wpi). But, at the sixth PS injection (at 5 wpi), mice in the two control groups (HBV + PS, PBS; PS, PBS) were injected with PBS (i. p.) instead of PS, and then at the indicated time points within 72 h after the PS (PBS) injection, the HBsAg, ALT, and cytokines were tracked.

Blood samples were collected from the submandibular vein of mice (under isoflurane anesthesia), and then centrifuged (3,500 rpm) for 30 min to obtain serum. The left lobes of livers were fixed for preparation of paraffin sections, while a part of the right lobe of livers was used to isolate hepatic mononuclear cells, and the rest was frozen at −80℃ for later use.

### ETV treatment

ETV was used to inhibit HBV replication. Mice were randomly divided into three groups (6 mice per group), including PBS, Model, and ETV, based on their serum levels of HBsAg at 6 wpi. Mice in groups of Model and ETV were injected both with pAAV-HBV1.2 plasmid (HI) and PS (i.p.), the same as the protocol shown in Fig. 1A, whereas those in the PBS group were substituted with injections of the same volume of PBS, respectively, for the plasmid or PS. ETV (suspended in 0.5% CMC-Na) was intragastrically given to the mice in the ETV group at a dose of 0.1 mg/kg daily for 6 weeks (from 6 wpi). Mice in PBS and Model groups were administered with the corresponding volume of 0.5% CMC-Na.

### ELISA

Levels of HBsAg, HBeAg, and HBsAb in mouse serum were measured by the enzyme-linked immunosorbent assay (ELISA) method. The cytokines were assayed according to the corresponding instructions of the kits from MultiSciences (Lianke) Biotech Co., Ltd., Hangzhou, China. HBsAg was quantified by using an ELISA kit (Elabscience Biotechnology Co., Ltd., Wuhan, China). HBeAg and HBsAb were examined using kits obtained from Shanghai Kehua Bioengineering Co., Ltd. (Shanghai, China).

### Quantitative real-time PCR

HBV DNA was extracted from 50 µL serum samples and then detected by quantitative real-time PCR (qPCR) using an HBV nucleic acid diagnostic kit according to the instructions provided by the manufacturer (Shanghai Kehua Bioengineering Co. Ltd).

### H&E and Sirius red staining

As previously described (38), briefly, paraffin-embedded hepatic specimens were sliced into 3 µm (H&E) or 4 µm (Sirius red) thick sections using a RM2245 rotary microtome (Leica, Germany). Then, the sections were dewaxed in xylene, rehydrated in ethanol, and stained with H&E for histological evaluation and stained with Sirius red for collagen fibers. The level of LF was evaluated by calculating the percentage of the red region (collagen) by Image-Pro-Plus software (version 6.0). Inflammation was assessed according to the Scheuer scoring system. The stage of LF was appraised following the Ishak scoring system.

### IHC staining

Liver sections (3 µm) were incubated in 3% hydrogen peroxide for 30 min followed by a PBS wash. Then, the sections were blocked with 3% fetal bovine serum at room temperature following heat-mediated antigen retrieval and incubated successively with primary antibodies against α-SMA rabbit polyclonal antibody (1:800; Proteintech Group, Inc., Wuhan, China), HBcAg (1:50; Gene Tech [Shanghai] Company Limited), or PD-L1 (1:100; HUABIO, Huaan Biotechnology Co., Ltd., Hangzhou, China), at 4℃ overnight, and secondary antibody (1:300; Thermo Fisher Scientific [China] Co., Ltd.) at 37℃ for 45 min, and then treated with a diaminobenzidine chromogenic substrate, counterstained with hematoxylin, sealed, and scanned with an Aperio Versa scanner (Leica, Germany). The protein expression levels of α-SMA and HBc were semi-quantified with IHC scores (38). The final IHC score was obtained using the score for the percentage of positive-stained cells multiplied by the score for staining intensity. In brief, the scores of staining intensities were defined as: score 0 for negative staining, score 1 for weak staining, score 2 for moderate staining, and score 3 for strong staining. The scores for positive-stained cells were set as follows: score 1 for <25% of positive cells; score 2 for 25%–50% of positive cells; score 3 for 50%–75% of positive cells; and score 4 for 75%–100% of positive cells.

### Western blotting

Protein samples were obtained by lysing liver tissues with RIPA lysis buffer (Beyotime, Shanghai, China). After the equal amounts of proteins were separated on a 10% SDS-PAGE gel, the proteins were transferred onto a nitrocellulose (NC) membrane. Membranes were blocked with 5% skim milk powder for 1 h at room temperature, then incubated with diluted primary antibodies against α-SMA (1:2,000; Proteintech Group, Inc., Wuhan, China), HBc (1:1,000, Abcam, Cambridge, UK), or β-actin (1:2,000, Proteintech Group, Inc., Wuhan, China) at 4℃ overnight, followed by incubation with the secondary antibody (1:4,000, Thermo Fisher Scientific (China) Co., Ltd., Shanghai, China) for 1 h, and then the target bands were visualized using a chemiluminescence imager (C300, Azure Biosystem Inc., Dublin, OH, USA). The western blot band intensities were quantified using Image J 1.8.0.

### Measurements of hydroxyproline and ALT

The hepatic hydroxyproline and the serum ALT were separately analyzed via a hydroxyproline assay kit (A030-2-1) and an alanine aminotransferase assay kit (C009-2-1) obtained from Nanjing Jiancheng Bioengineering Institute (Nanjing, China).

### Isolation of hepatic mononuclear cells and flow cytometry

The right lobes of mouse livers were removed, washed with PBS, and pressed through a 200-gauge mesh. The cells were then suspended in 3 mL of 40% Percoll solution, gently overlaid onto 2 mL of 70% Percoll solution (Gentihold, Beijing JintaiHongda Biotechnology Co., Ltd., Beijing, China), and centrifuged (1,600 rpm, 30 min) at 4℃. The hepatic mononuclear cells at the interface between the two Percoll solutions were collected and suspended in 5 mL of 40% Percoll solution.

Cells were isolated and directly incubated with anti-CD3 PerCP-eFluor 710 monoclonal antibody (46-0037-42; eBioscience, San Diego, CA, USA), anti-CD8a APC monoclonal antibody (17-0081-81; eBioscience, San Diego, CA, USA), and anti-PD-1 PE monoclonal antibody (12-9985-81; eBioscience, San Diego, CA, USA), then washed with PBS. The fluorescence data were acquired on a BD LSRFortessa Flow Cytometer (BD Bioscience, USA) and then analyzed using FlowJo 10.0 (BD Bioscience, USA).

### Statistical analysis

All statistical analyses were performed using SPSS version 25.0 (SPSS Inc., Chicago, IL, USA). The two independent samples *t*-test, one-way analysis of variance (ANOVA), and Kruskal-Wallis test were employed. *P* < 0.05 was considered significant.

## Data Availability

The data during the current study are available from the corresponding author on reasonable request.

## SUPPLEMENTAL MATERIAL

Figure S1 spectrum.02513-24-s0001.pdf Lack of differences in LF progression among groups of C57BL/6J mice (12 wpi).

Supplemental material spectrum.02513-24-s0002.doc Materials and software.

ASM does not own the copyrights to Supplemental Material that may be linked to, or accessed through, an article. The authors have granted ASM a non-exclusive, world-wide license to publish the Supplemental Material files. Please contact the corresponding author directly for reuse.
