# Supplementary material for: A novel chronic hepatitis B mouse model with immune activation and liver fibrosis
Source: Microbiol Spectr. 2025 Jul 24;13(9):e02513-24. doi: 10.1128/spectrum.02513-24 (PMC12419765; doi:10.1128/spectrum.02513-24)
Supplement: Figure S1 — Lack of differences in LF progression among groups of C57BL/6J mice (12 wpi). [file spectrum.02513-24-s0001.pdf]

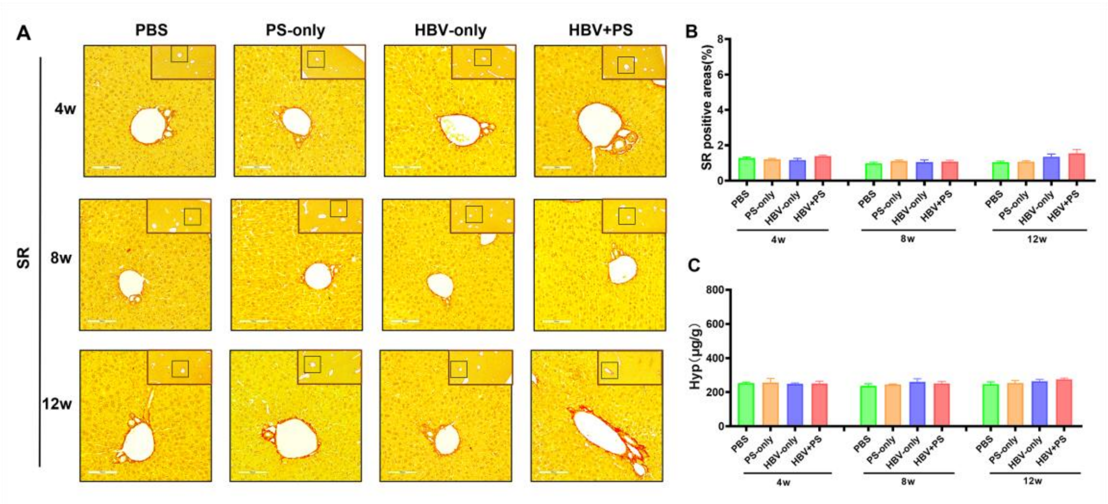

FIG S1 Lack of differences in LF progression among groups of C57BL/6J mice (12 wpi). Levels of collagen deposition were appraised by (A, B) Sirius red (SR) staining of liver sections, and by (C) the quantification of hepatic hydroxyproline (Hyp, n=6); for the representative images of SR staining, 200×, scale bar=100 μm; top right corner, 100×; scale bar=200 μm.
