# Supplementary material for: A novel chronic hepatitis B mouse model with immune activation and liver fibrosis
Source: Microbiol Spectr. 2025 Jul 24;13(9):e02513-24. doi: 10.1128/spectrum.02513-24 (PMC12419765; doi:10.1128/spectrum.02513-24)
Supplement: Supplemental material — Materials and software. [file spectrum.02513-24-s0002.doc]

# MATERIALS AND SOFTWARE

| **MATERIALS** | **SOURCE** | **CATLOG** |
| --- | --- | --- |
| plasmid |  |  |
| pAAV-HBV1.2 | Prof.Pei-Jer Chen (National Taiwan University, Taipei, China) | Cat # Non-applicable |
| Chemicals, peptides, and recombinant proteins |  |  |
| entecavir (ETV) | Chia Tai Tianqing Pharmaceutical Group Co., Ltd. | H20100019 |
| porcine serum | Gibco | Cat #26250084 |
| carboxymethylcellulose sodium (CMC-Na) | Anhui Sunhere Pharmaceutical Excipients Co., Ltd. | Cat #SH-SJJ-2000 |
| Percoll | Gentihold | Cat #P1068 |
| Critical commercial assays |  |  |
| Hepatitis B virus surface antigen ELISA Kit | Elabscience Biotechnology Co., Ltd | Cat #E-EL-H6080 |
| Hepatitis B virus surface antibody ELISA Kit | Kehua Bioengineering Co. Ltd. | Cat #A096999 |
| Hepatitis B e antigen ELISA Kit | Kehua Bioengineering Co. Ltd. | Cat #A097002 |
| HBV DNA Assay Kitwas obtained from | Kehua Bioengineering Co. Ltd. | Cat #20163401197 |
| Alanine aminotransferase assay kit | Nanjing Jiancheng Bioengineering Institute | Cat #C009-2-1 |
| Hydroxyproline assay kit | Nanjing Jiancheng Bioengineering Institute | Cat #A030-2-1 |
| TNF-αELISA kits | MultiSciences | Cat #EK282 |
| IFN-γELISA kits | MultiSciences | Cat #EK280 |
| IL-1βELISA kits | MultiSciences | Cat #EK201B |
| Antibodies |  |  |
| Anti-HBcAgprimary antibody | Gene Tech | GB058621/29/11 |
| Anti-HBcAg primary antibody | Abcam | Ab8637 |
| Anti-α-SMA rabbit polyclonal antibody | Proteintech | Cat #80008-1-RR |
| Anti-PD-L1 Recombinant Rabbit Monoclonal Antibody | Huabio | Cat #HA722184 |
| Goat anti-rabbit IgG secondary antibody | Thermo Fisher | Cat #31460 |
| Goat anti-mouse IgG secondary antibody | Thermo Fisher | Cat #31800 |
| Anti-CD3 PerCP-eFluor 710monoclonal antibody | eBioscience | Cat #46-0037-42 |
| Anti-CD8a APC monoclonal antibody | eBioscience | Cat #17- 0081-81 |
| Anti-PD-1 PE monoclonal antibody | eBioscience | Cat #12-9985-81 |
| Software and algorithms |  |  |
| FlowJo™ software v10.0.7 | Becton Dickinson Bioscience | Cat # Non-applicable |
| Image J 1.8.0 | National Institutes of Health | Cat # Non-applicable |
| GraphPad Prism 8.0.1 | Graphpad Software, Inc | Cat # Non-applicable |
| IBM© SPSS© 25 Statistics software | IBM Corporation | Cat # Non-applicable |
